# Mix Design and Early-Age Performance of Rapid-Setting Phosphate-Based CBPCs for Emergency Road Repair

**DOI:** 10.3390/ma18174045

**Published:** 2025-08-29

**Authors:** Jaeyoung Lee

**Affiliations:** Department of Fire Administration and Disaster Management, The College of Humanities & Social Sciences, Dong-Eui University, Busan 47340, Republic of Korea; lee_jy@deu.ac.kr

**Keywords:** emergency road repair, Chemically Bonded Phosphate Ceramics (CBPCs), rapid-setting materials, mechanical properties

## Abstract

This study investigates rapid-setting, phosphate-based, chemically bonded phosphate ceramic (CBPC) composites for emergency pothole repair through a two-phase experimental approach. Phase I involved fundamental mix design experiments that systematically examined the effects of water-to-binder ratio (20–40%), filler content (10–50%), and phosphate powder fineness (570–3640 cm^2^/g) on setting and mechanical performance. Based on Phase I results, Phase II evaluated field-applicable mixes optimized for concrete and asphalt pavement conditions in terms of rapid strength development: compressive strength exceeding 24 MPa within 30 min, flexural strength surpassing 3.4 MPa within 1 h, and adhesive strength reaching up to 1.62 MPa (concrete) and 0.68 MPa (asphalt) within 4 h. Additional performance evaluations included Marshall stability (49,848 N), water-immersion residual stability (100% under the test protocol), length change (small magnitude over 28 days), and self-filling behavior (complete filling in 17 s in the specified setup). These rapid early-age results met or surpassed relevant domestic specifications used for emergency repair materials. Based on these data, mix designs for field application are proposed, and practical implications and limitations for early-age performance are discussed.

## 1. Introduction

Asphalt pavements are generally reported to have a service life of approximately 10 years [1]; however, actual longevity can vary significantly depending on environmental conditions and traffic loads [2,3,4,5]. In recent decades, the incidence of potholes has sharply increased, posing significant safety risks as potholes that exceed certain dimensions in diameter and depth can lead to major traffic accidents [6,7]. Pothole formation is influenced by multiple factors, including the presence of water, mechanical loading, and temperature variations [2,8]. Two primary mechanisms have been identified: surface deterioration initiated by water infiltration and traffic loads through cracks in the pavement surface, resulting in localized pothole development and subgrade erosion caused by water penetration, leading to loss of support beneath the asphalt layer and subsequent structural failure. Given these safety and performance risks, the timely and durable restoration of potholes has become a critical need for pavement agencies.

Currently, emergency repairs predominantly use cold-mix asphalt materials; however, these materials tend to deteriorate within 6 to 7 months and exhibit aging mechanisms similar to those of existing pavements, often causing secondary damage around the repair area. Therefore, materials with rapid setting, excellent adhesion, and stability are required for the rapid repair of potholes on asphalt roads [9].

Chemically Bonded Phosphate Ceramics (CBPCs) composites are inorganic materials formed through a chemical reaction between phosphate and magnesium oxide (MgO) and are considered suitable for pothole repair due to their ultra-rapid hardening and high strength. Existing research has demonstrated the advantages of CBPCs for road repair, including their ability to achieve early strength and durability under various conditions. For example, according to previous studies, CBPCs have been reported to exhibit superior performance in terms of setting time and strength development compared to conventional materials [10,11,12,13].

As a benchmark retarder in MPC/MKPC systems, borax has been shown to delay setting while improving workability with a known trade-off in early strength; recent work using Mg-slag–derived MPC confirms this functional role and microstructural control mechanism, supporting our use of 1% borax as the baseline retarder in this study. In brief, under typical Magnesium Potassium Phosphate Cement (MKPC) conditions, struvite-K (KMgPO_4_·6H_2_O) is the principal binding phase governing early strength, and borate species moderate early hydration kinetics; detailed mechanistic pathways are discussed in the background section [14,15,16]. Complementary research has identified zinc acetate as a potent alternative set retarder for MKPC, enabling targeted control of setting time but with concomitant impacts on hydration kinetics and early mechanical development; given our emphasis on rapid early strength and constructability for emergency repair, we prioritized borax-based mixes over zinc-acetate systems [17].

Accordingly, this study tests the hypothesis that optimizing water-to-binder ratio (W/B), filler content, and phosphate powder fineness within borax-retarded CBPCs can deliver sub-hour structural strength and robust constructability required for emergency pothole repair, while meeting domestic specifications. The rheological response of MPC–emulsified asphalt composites is highly sensitive to MgO-to-phosphate (M/P) ratio and processing parameters, indicating that mix design choices directly govern workability, dispersion stability, and early-age performance; these insights motivate our systematic evaluation of W/B, filler up to 50%, and phosphate fineness for field-applicable CBPC formulations targeting asphalt and concrete pavements. Taken together, prior evidence on borax-mediated setting control and the trade-offs of stronger zinc-based retarders, combined with rheology–constructability coupling in MPC–asphalt composites, provides the rationale for our mix-design matrix and our focus on early-age strength, adhesion, stability, and self-filling under realistic repair conditions [17,18,19].

Nevertheless, despite these advances, most previous studies have centered on optimizing the basic mix design of CBPC composites, often overlooking several key factors that affect field applicability. Specifically, there has been insufficient systematic analysis of the effects of filler (sand) incorporation rate, phosphate powder fineness, and the diversity of material components on the performance of CBPCs in real-world repair scenarios. Additionally, comprehensive performance evaluations—including adhesive strength, self-filling ability, and shrinkage rate—have rarely been conducted, leaving gaps in the understanding of how these materials behave under practical field conditions [20,21].

To address these limitations, this study provides a thorough analysis of the hardening and mechanical properties of CBPC composites under expanded mix conditions. We investigate the feasibility of incorporating up to 50% filler, examine the influence of phosphate powder fineness, and develop mix designs tailored for field application, with a focus on compressive strength, flexural strength, adhesion strength, stability, and other critical performance indicators. A series of laboratory experiments and field application evaluation tests were conducted to evaluate these variables systematically and to establish robust guidelines for the practical use of CBPCs in emergency road repair.

## 2. Experimental Plan and Methods

**Figure 1:**
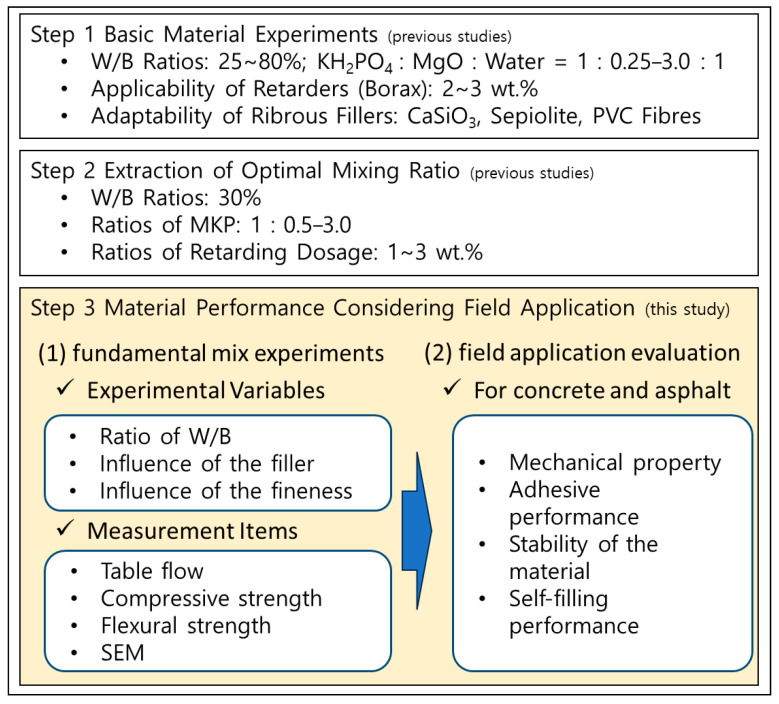
Overview of the research framework and experimental plan.

Figure 1 illustrates the overall research framework adopted in this study, from the fundamental mix design phase—drawing on prior findings by Lee and Kim [20,21]—to the field performance evaluation phase conducted herein. The first phase comprised systematic variation of key mix parameters, namely, the water-to-binder (W/B) ratio (20–40%), filler (sand) content (10–50%), and phosphate powder fineness (570–3640 cm^2^/g), to assess their effects on setting time and strength development.

The second phase focused on evaluating the field applicability of borax-retarded CBPC composites by determining optimal mix proportions for concrete and asphalt pavements and measuring mechanical properties, adhesion, stability, and self-filling ability in accordance with the Ministry of Land, Infrastructure and Transport standards [22].

### 2.1. Experimental Plan and Measurement Items

Experiments were designed in two main parts: (1) fundamental material mix experiments to analyze the hardening and mechanical properties under various mix conditions (hereinafter referred to as “fundamental mix experiments”), and (2) performance evaluation of the optimal mix for field application (hereinafter referred to as “field application evaluation”). Table 1 summarizes the mix conditions adopted for the fundamental mix experiments.

In Test 1, particular attention was given to the effects of the W/B ratio, filler (sand) incorporation rate, and phosphate powder fineness, as previous studies by Lee and Kim [20,21] had not sufficiently addressed these variables.

In Test 2, the mixing ratio of phosphate (P) to magnesium oxide (M) was fixed at 1:1.5, and the retarder (borax) was set at 1%. The W/B ratio was adjusted to five levels (20–40%). In Test 2, manufactured sand (No. 5 Sand) was used as the filler, and the influence of the filler incorporation rate on material performance was analyzed. This variable plays a critical role in ensuring economic feasibility.

In Test 3, to investigate the effect of phosphate powder fineness on material performance, the grinding unit of the fine mill was adjusted to three levels (0 cm [no processing], 2.8 cm, and 3.7 cm), resulting in potassium dihydrogen phosphate powder with fineness values of 570, 2970, and 3640 cm^2^/g, respectively.

The measurement items for each mix included flowability and setting time (initial hardening time) immediately after mixing; compressive and flexural strengths over time; adhesive strength; surface finish; and internal microstructure observations via SEM imaging.

All experiments were conducted in accordance with KS (Korean Industrial Standards), and measurements were taken at eight ages—15 min, 30 min, 60 min, 1 day, 3 days, 7 days, 14 days, and 28 days—to capture changes in material properties at early and intermediate stages.

In the field-application evaluation, the optimal mix for field use was determined based on the results of the fundamental mix experiments. For the final mix, a comprehensive assessment was performed, including compressive strength, flexural strength, adhesive strength, self-filling ability, stability, and length change.

### 2.2. Materials Used and Specimen Preparation

In this study, potassium dihydrogen phosphate (KH_2_PO_4_) was adopted as the phosphate, consistent with Lee and Kim [20,21], and dead-burned magnesium oxide (Dead Burn MgO) was used as the source of magnesium oxide. Borax (Na_2_B_4_O_7_·10H_2_O) was applied as a retarder. The chemical composition and physical properties of each material are summarized in Table 2, Table 3, Table 4 and Table 5. The artificial sand used in this study is an aggregate obtained by crushing granite. As shown in Table 5, it has a higher SiO_2_ content compared to other sands and consequently exhibits relatively higher particle strength (%).

Specimen preparation involved dry mixing the powdered materials for 30 s according to the mix proportions, followed by wet mixing with water for 3 min. Subsequently, cube-shaped specimens measuring 50 × 50 × 50 mm (for compressive strength testing) and prism-shaped specimens measuring 40 × 40 × 160 mm (for flexural strength testing and length change) were prepared. Specimens were air-cured at 20 ± 3 °C and 55 ± 5% RH; unless noted, tests were conducted under the same environment. As a preliminary study, the number of specimens per condition was limited to one (n = 1) due to resource constraints and the exploratory scope. We recognize this limitation and will include replicates and statistical validation in subsequent work.

### 2.3. Measurement Method

Specimens were air-cured at 20 ± 3 °C and 55 ± 5% RH unless otherwise noted, and all tests were performed in a temperature- and humidity-controlled chamber. Flowability was measured using a flow table with 25 drops, reporting the average of two orthogonal diameters, in accordance with KS L 5111 [23]. Compressive strength was tested on 50 × 50 × 50 mm cubes at a loading rate of 0.6 ± 0.4 MPa/s, following KS L 5105 [24], and flexural strength was measured on 40 × 40 × 160 mm prisms using a three-point bending jig with a 100 mm span at 50 N/s, in accordance with KS F 2408 [25]. Compressive strength and flexural strength were measured using a universal testing machine (Shimadzu Autograph AGS-X series, Shimadzu Corporation, Kyoto, Japan). Adhesive strength (pull-off) was evaluated by casting CBPCs into 50 × 50 × 10 mm molds placed on concrete and asphalt substrates (each 300 × 300 × 50 mm) per KS F 4042 [26]; the concrete substrate (50 MPa) was ground to remove laitance and brushed, while the asphalt substrate received a thin epoxy bonding layer; circular dollies (Ø50 mm) were bonded and tested at 0.05 MPa/s, and maximum adhesive stress and failure modes were recorded. Length change was measured on 40 × 40 × 160 mm prismatic specimens using a Demec mechanical strain gauge (MIC-810-0-02, Marui Co., Ltd., Nagoya, Japan) in accordance with KS F 2424 [27].

## 3. Mechanical Properties Based on Material Mix Design

### 3.1. Hardening and Mechanical Properties by W/B Ratio

**Figure 2:**
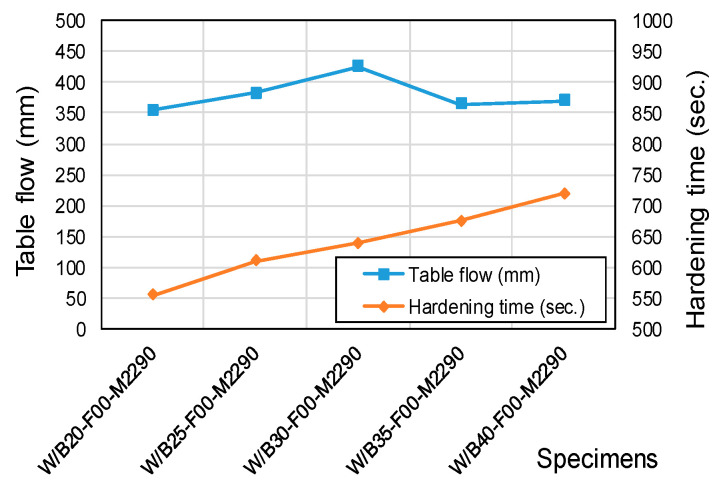
Flowability and setting time as a function of W/B ratio.

Figure 2 shows the variation in flowability and setting time as the W/B ratio increases. Flowability exhibited a linear increase up to a W/B ratio of 30%, but declined by 14.3% (from 425 mm to 370 mm) when the W/B ratio was raised to 35–40%. The decrease in flowability has been explained in previous studies as being related to delayed aggregation of magnesium potassium phosphate (MKP)—the reaction product of KH_2_PO_4_ and MgO—which occurs when the water content is excessive [28]. The setting time increased by approximately 7.9 s for each 1% increment in the W/B ratio, which can be interpreted as a retardation of the neutralization reaction due to the dilution of Mg^2+^ ions. This observation is consistent with the findings of Dabarera et al. [29].

**Figure 3:**
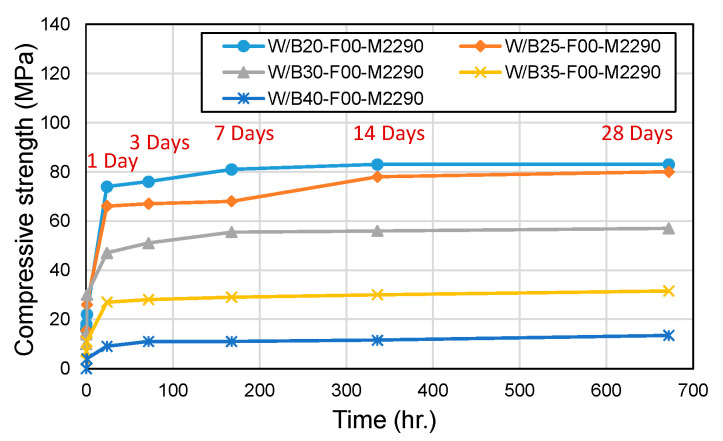
Compressive strength with W/B ratio.

**Figure 4:**
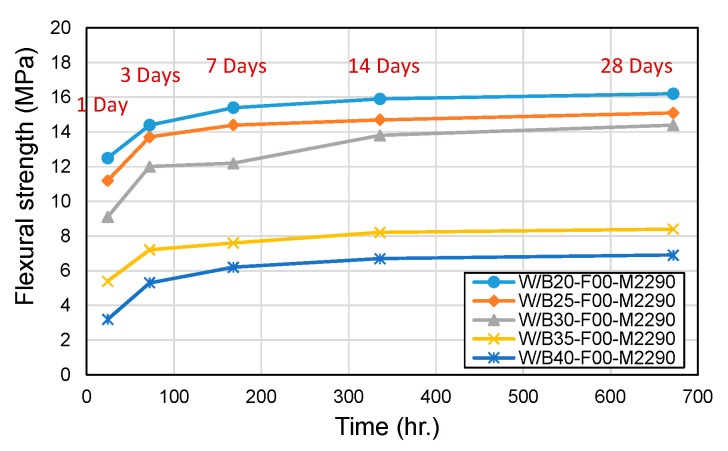
Flexural strength with W/B ratio.

Figure 3 and Figure 4 present the results for compressive and flexural strength, respectively. At W/B ratios of 20% and 25%, the 28-day compressive strength reached approximately 80 MPa. In contrast, higher W/B ratios (30%, 35%, and 40%) resulted in compressive strengths below 60 MPa, which is likely attributable to increased porosity and the presence of unreacted MgO, both of which contribute to structural defects. While Lee and Kim [20,21] suggested that a W/B ratio of 30% is optimal, the results of this study indicate that W/B ratios in the range of 20–25% should be considered for applications that require high compressive strength (>50 MPa). The flexural strength was found to be 19–27% of the compressive strength, which is consistent with the trends observed in conventional concrete. However, at higher W/B ratios (40%), the flexural strength ratio decreased significantly, with W/B40 showing a notably reduced flexural-to-compressive strength ratio, indicating deteriorated mechanical performance at excessive water contents.

### 3.2. Hardening and Mechanical Properties by Filler Incorporation

**Figure 5:**
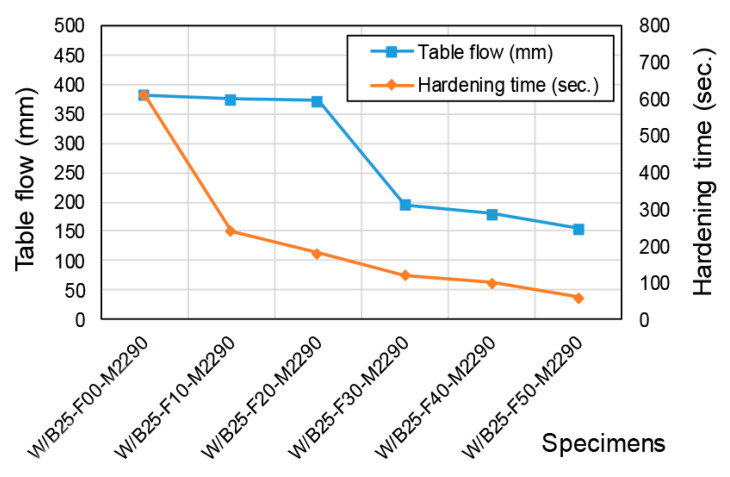
Flowability and setting time as a function of filler content.

Figure 5 illustrates the impact of filler incorporation on flowability. At a 20% filler incorporation rate, no substantial change in flowability was observed. However, increasing the rate to 30% resulted in a marked reduction in flowability (372 mm → 195 mm). This decline is attributed to diminished formation of CBPC reaction products, such as magnesium potassium phosphate (MKP), which inherently possess high flowability, coupled with enhanced interlocking effects from the sand filler. Regarding setting time, even a 10% filler incorporation significantly shortened the hardening duration. Beyond 10%, further increases in the incorporation rate progressively reduced the setting time.

**Figure 6:**
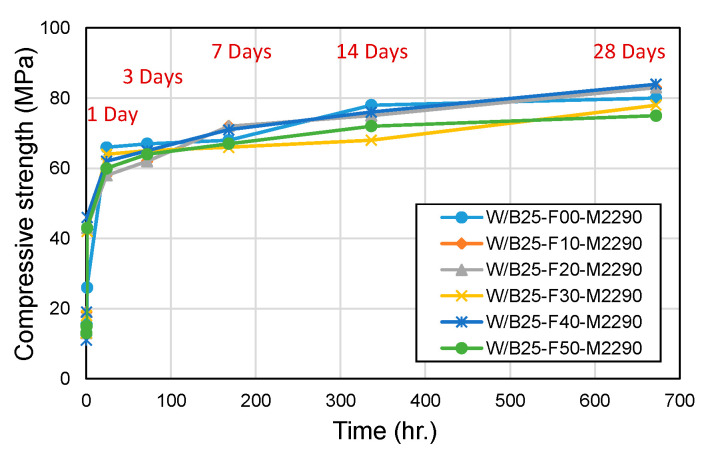
Compressive strength with filler content.

**Figure 7:**
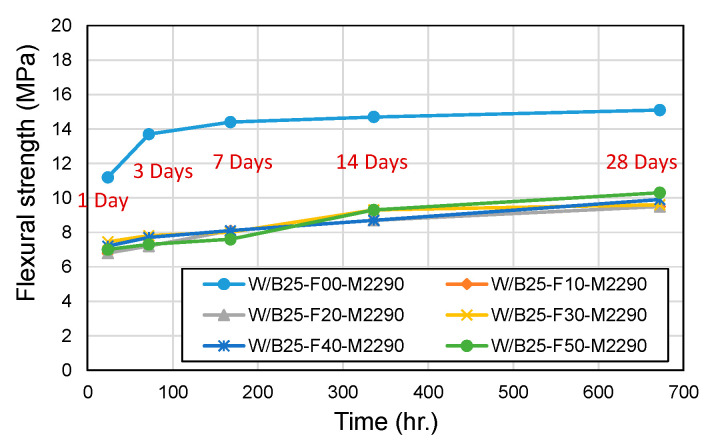
Flexural strength with filler content.

Figure 6 and Figure 7 compare compressive and flexural strength across filler incorporation rates. The 24 h compressive strength showed a slight overall decrease, ranging from 83% to 106% of the strength measured in filler-free mixes. In contrast, flexural strength exhibited a significant decline due to filler incorporation, dropping to 51–68% of the control values, demonstrating a stronger sensitivity to filler content. In the filler-free condition, the flexural strength represented approximately 19% of the compressive strength. However, with filler incorporation, the flexural strength decreased substantially, and the relative ratio to compressive strength also dropped to approximately 11–14% levels. These findings underscore the trade-offs between workability and mechanical performance in filler-incorporated CBPC systems, with implications for optimizing formulations in applications requiring rapid strength development, such as road repair.

### 3.3. Hardening and Mechanical Properties by Phosphate Powder Fineness

**Figure 8:**
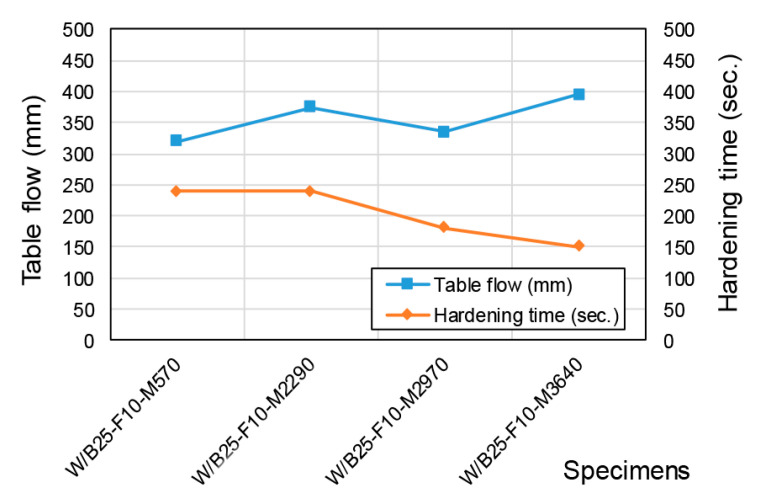
Flowability and setting time as a function of phosphate powder fineness.

Figure 8 presents the changes in flowability and setting time with respect to phosphate powder fineness. Flowability improved as fineness increased, which is attributed to the greater surface area of finer particles, resulting in enhanced reactivity with magnesium and promoting the formation of viscous reaction products. Quantitatively, the table flow increased with fineness (570 → 2970 → 3640 cm^2^/g) from 320 mm to 335 mm to 395 mm (Figure 8), corroborating the observed trend despite the common intuition that finer particles may increase water demand. Setting time gradually decreased with increasing fineness, indicating that the accelerated hydration was caused by faster magnesium-phosphate reactions. The W/B25-F10-M2290 specimen, which employed commercially processed phosphate powder, exhibited relatively high flowability but a longer setting time. This suggests that material characteristics influenced by processing methods, in addition to fineness itself, play a significant role.

**Figure 9:**
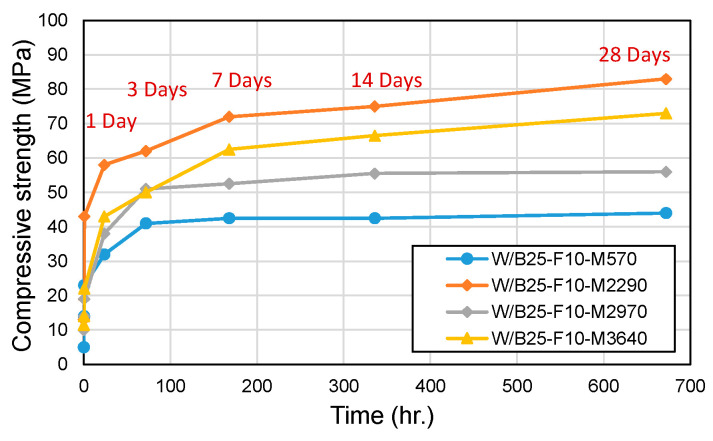
Compressive strength with phosphate powder fineness.

**Figure 10:**
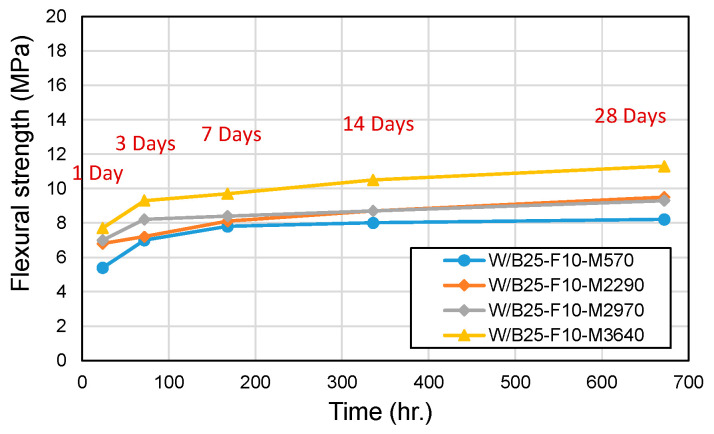
Flexural strength with phosphate powder fineness.

Figure 9 and Figure 10 present compressive and flexural strength results. Compressive strength showed similar values across fineness levels within the first 60 min. However, at 28 days, compressive strengths varied significantly (73 MPa, 56 MPa, 73 MPa, and 44 MPa). Notably, even unprocessed phosphate powders achieved compressive strengths exceeding the 40 MPa threshold required for road pothole repair materials. Flexural strength in standard mixes (excluding the processed W/B25-F10-M2290) ranged between 15 and 19% of compressive strength. The processed W/B25-F10-M2290 mix, despite its higher compressive strength, exhibited lower flexural strength (11% of compressive strength), likely due to microstructural changes from processing that increased brittleness. These findings indicate that while higher phosphate powder fineness improves flowability and setting speed, highly processed phosphate powders are not strictly necessary for practical applications.

### 3.4. Surface Properties and SEM

**Figure 11:**
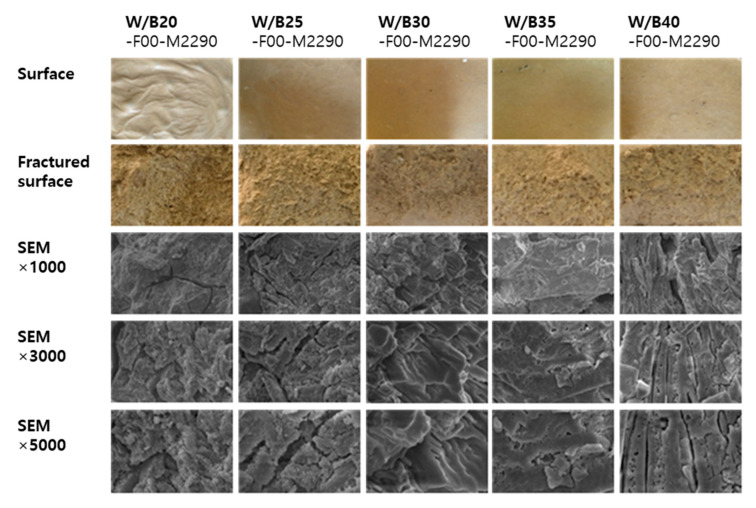
Surface appearance, fractured cross-section, and SEM images of the specimens according to different W/B ratios.

**Figure 12:**
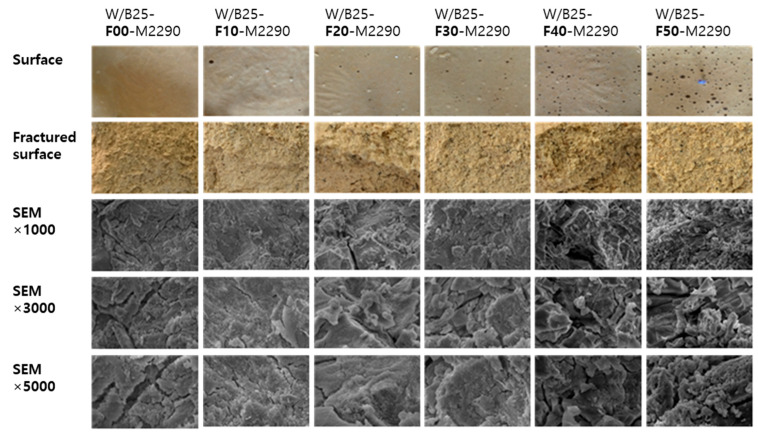
Surface appearance, fractured cross-section, and SEM images of the specimens with varying filler contents.

Figure 11 presents surface images, fractured surfaces after the compression test, and cross-sectional SEM (Scanning Electron Microscope) images of specimens with varying W/B ratios (20–40%). Figure 12 shows the corresponding images for specimens with different filler contents (10–50%). The surface condition of the mixtures without filler appeared to be favorable at W/B ratios of 30–40%. In the SEM images, the plate-like structure became more distinct as the W/B ratio increased. When varying the filler content in mixtures with a W/B ratio of 25%, the number of surface pores increased with higher filler content. No significant changes were observed in the SEM images according to the addition of filler.

## 4. Performance Evaluation Experiments for Field Application

### 4.1. Material Mix Design and Evaluation Items Considering Field Application

**Figure 13:**
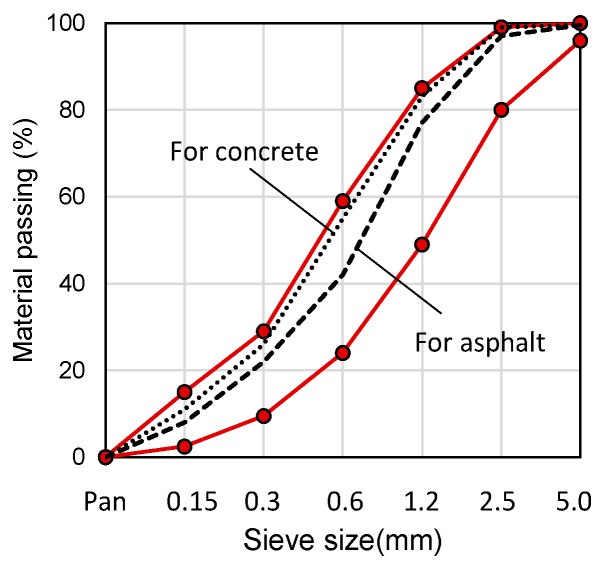
Particle size distribution (fineness modulus) of aggregates for concrete and asphalt mixes.

The mix proportions for field application, specifically the W/B ratio, were determined based on the experimental results of Lee and Kim [20,21] and those presented in Section 3 of this study. In addition, it was taken into consideration that domestic road finishing materials are primarily concrete and asphalt. The filler content was set at 50%, as increasing the filler ratio did not significantly decrease the strength. The mix conditions suitable for each road type are detailed in Table 6. As fillers, artificial silica sands No. 3 Sand, No. 5 Sand, No. 6 Sand, and Stone Powder were appropriately mixed and used at a 50% ratio. The particle size distribution test was conducted according to KS F 2502 (similar to ASTM C136) [30], and the results are shown in Figure 13. It was confirmed that the packing densities of coarse aggregates for both concrete and asphalt pavements fall within the standard ranges specified by KS F 2527 (similar to ASTM C33/C33M) [31].

The evaluation items for the material mix for field application included compressive strength, flexural strength, adhesive strength, stability, water-immersion residual stability, shrinkage rate, and self-filling ability.

However, this study did not include long-term durability evaluation. Stability, water-immersion residual stability, shrinkage rate, and self-filling ability evaluations were conducted by arbitrarily selecting commercially available grout products for comparative evaluation. Stability and water-immersion residual stability evaluations were performed according to KS F 2409 standards (similar to ASTM D6927) [32,33].

### 4.2. Mechanical Properties

**Figure 14:**
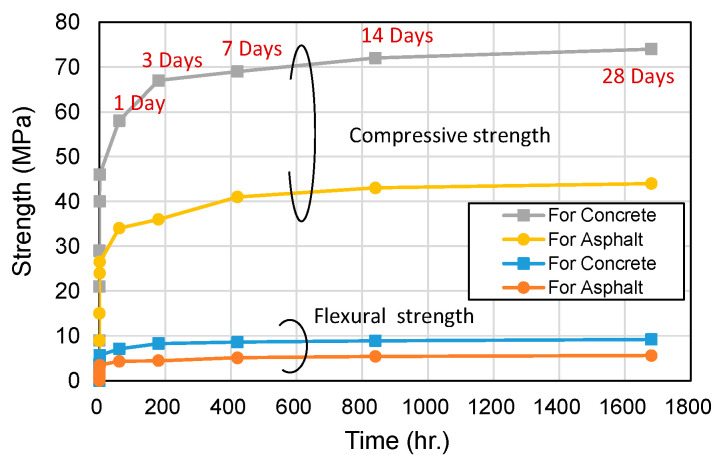
Compressive and flexural strength development for concrete and asphalt mixes.

Optimized CBPC mixes were applied based on the base conditions of the road (concrete or asphalt). Changes in compressive and flexural strengths over time after placement are shown in Figure 14. For concrete-type CBPCs, the 28-day compressive strength was measured at 74 MPa, while asphalt-type CBPCs achieved 44 MPa. Both mixes significantly exceeded the 28-day compressive strength standard (≥40 MPa) required for road construction. Notably, both mixes attained compressive strengths exceeding 24 MPa within just 30 min of placement, satisfying the road construction standard (≥21 MPa after 4 h of open time) in a remarkably short period.

Flexural strength was approximately 12% of compressive strength, measured at 9.2 MPa for concrete-type CBPCs and 5.6 MPa for asphalt-type CBPCs. Both mixes demonstrated flexural strengths surpassing 3.4 MPa within one hour of placement, meeting the road construction standard (≥3.15 MPa after 4 h of open time) at an early stage. These results confirm that the CBPC composite exhibits excellent early strength development and durability as an emergency road repair material.

Concrete-type CBPCs showed approximately 30 MPa higher long-term compressive strength compared to asphalt-type CBPCs, making them suitable for high-strength applications in special sections such as bridges and tunnels. Asphalt-type CBPCs, while meeting all strength standards, also demonstrated economic advantages due to their cost-effectiveness.

The CBPC mixes proposed in this study satisfy road construction quality standards early, ensuring both field applicability and reliability.

**Figure 15:**
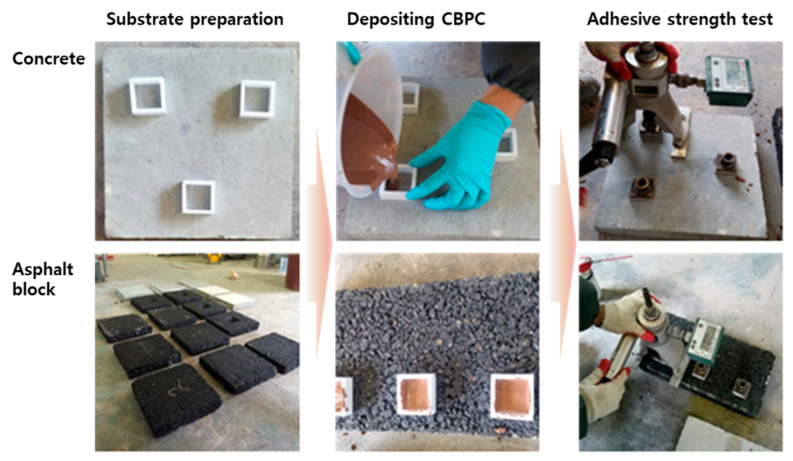
Procedure for adhesive strength testing (Note: asphalt specimens employed a thin epoxy primer; concrete specimens did not).

The adhesive strength test was conducted by preparing specimens for both concrete (50 MPa, 300 × 300 × 50 mm) and asphalt, placing CBPCs on them, and measuring the adhesive strength. The asphalt specimens were fabricated by the Korea Conformity Laboratories (KCL). Figure 15 shows the process of placing CBPCs on each specimen and measuring the adhesive strength, in accordance with KS F 4042 standards (similar to EN 1542) [27].

**Figure 16:**
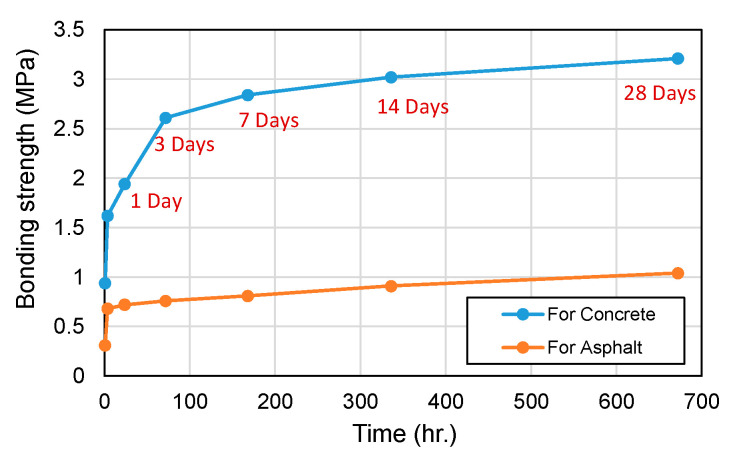
Adhesive strength development for concrete and asphalt mixes.

Figure 16 presents the adhesive strength results. In this study, a thin epoxy bonding layer was intentionally applied to the asphalt substrate prior to CBPC placement to enhance interfacial bonding; the concrete substrate was tested without epoxy. Consequently, the measured “adhesive strength” for the asphalt case reflects the CBPC–epoxy–asphalt system rather than a direct CBPC–asphalt interface.

For the concrete CBPCs, the 1 h adhesive strength was 0.94 MPa. For the asphalt case, 1 h and 4 h values were 0.31 MPa and 0.68 MPa, respectively, both exceeding the KS criterion of 0.24 MPa. The markedly low 1 h values—particularly on asphalt—are attributed primarily to incomplete curing of the pre-applied epoxy layer at the time of pull-off testing, rather than insufficient intrinsic adhesion of the CBPCs. After 4 h, as the epoxy progressed in curing, the measured strengths increased (e.g., concrete: 1.62 MPa, slightly above the KS criterion of 1.4 MPa), supporting this interpretation [27].

Note that the asphalt CBPC strengths remained lower than those of the concrete CBPCs, which is consistent with the organic nature and lower surface energy of asphalt mixtures; however, in the present protocol, the early-age measurements are constrained by the curing state of the epoxy primer. Future evaluations aimed at isolating the CBPC–substrate interface should (i) omit the epoxy primer or (ii) allow full primer curing per manufacturer specifications and report failure modes accordingly.

### 4.3. Stability and Shrinkage Rate

For Marshall stability and water-immersion residual stability tests, specimens were manufactured using dedicated molds. After compaction and demolding, the specimens were cured for 48 h under dry and water-immersion conditions at 25 °C. The Marshall stability of the CBPC repair material was measured at 49,848 N, significantly exceeding the standard for emergency repair cold asphalt mixtures (7350 N). The water-immersion residual stability was 100%, far surpassing the standard requirement of 75%.

**Figure 17:**
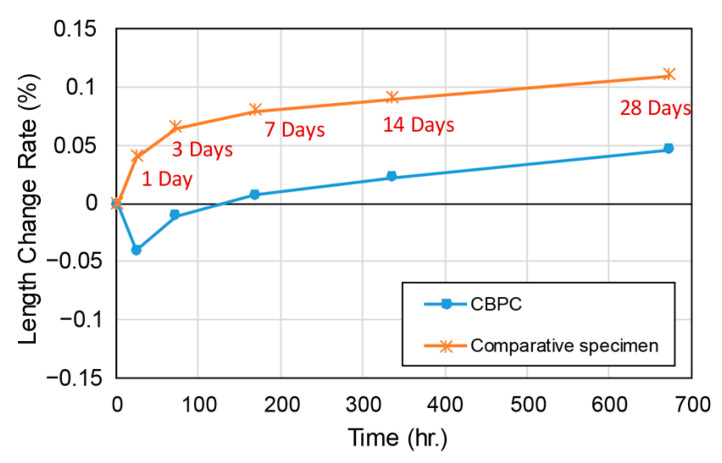
Length change rate of CBPC repair material and comparative specimen.

The length change of the CBPC repair material was measured using embedded shrinkage gauges immediately after placement, in accordance with KS F 2424 standards (similar to ASTM C157) [27]. Specimens were cured under dry conditions at 20 ± 3 °C and 40% relative humidity. Figure 17 shows the rate of length change: CBPC repair material initially exhibited a slight shrinkage of about −0.04% after 24 h, then gradually expanded, reaching approximately +0.047% at 28 days. This slight expansion is plausibly associated with the continued growth of struvite-K (KMgPO_4_·6H_2_O) and hydration-induced crystallization pressure under the given curing conditions. In comparison, the reference specimen showed expansion from the beginning, without shrinkage, and reached about +0.1% at 28 days.

These results indicate that the CBPC repair material demonstrates low risk of cracking and stable performance.

### 4.4. Self-Filling Ability

**Figure 18:**
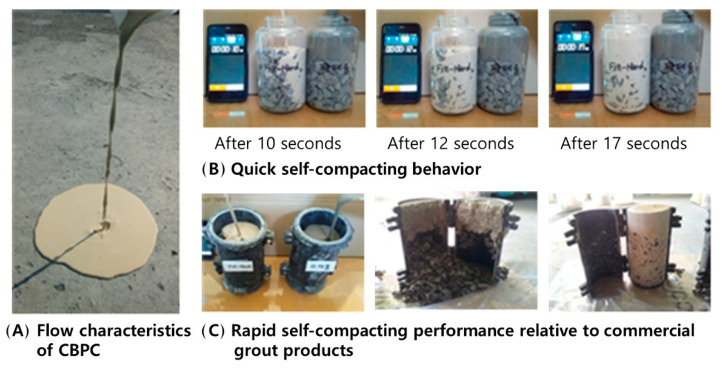
Self-filling properties of CBPCs and commercial grout.

To evaluate the self-filling ability of CBPCs, 13 mm coarse aggregate was placed in a specific container and a 10 × 20 cm compression mold. The filling state over time and the post-demolding morphology were compared with commercial grout (reference specimen). As shown in Figure 18, CBPCs demonstrated significantly higher self-filling ability than the commercial grout when coarse aggregate was placed in the container. CBPCs achieved complete filling within 17 s of placement, whereas the commercial grout failed to fully fill the gaps between aggregates.

In the 10 × 20 cm compression mold, CBPCs exhibited complete integration of aggregates and matrix after placement and demolding, achieving a fully filled state. In contrast, the commercial grout showed insufficient filling, leading to separation between aggregates and the matrix during demolding.

These results indicate that CBPCs can rapidly and uniformly fill voids under field conditions, demonstrating superior self-filling ability and strong interfacial adhesion.

## 5. Conclusions

Our experimental results showed that key mix parameters—including the W/B ratio, filler content, and phosphate powder fineness—significantly influenced the setting time and the development of compressive and flexural strengths. Notably, even with a 50% filler incorporation, the material maintained sufficient flowability and strength for field application. Furthermore, higher phosphate powder fineness was found to improve both flowability and setting speed.

Based on laboratory results, optimal mixes were selected for both concrete and asphalt pavement conditions. Comprehensive evaluations confirmed that all key performance indicators—including compressive strength, flexural strength, adhesive strength, dimensional stability, water-immersion durability, shrinkage behavior, and self-filling ability—exceeded the standards set by the Ministry of Land, Infrastructure and Transport. In particular, the developed CBPC composites demonstrated rapid early strength development, reaching structural strength levels within just 30 min of placement, and exhibited excellent self-filling ability, thereby enhancing on-site constructability and efficiency.

While the adhesive strength of the asphalt-type CBPCs was lower than that of the concrete-type, this difference is attributable to the organic nature of asphalt and does not significantly hinder its practical application.

The findings of this study suggest that CBPC composites can be widely applied in real-world road maintenance scenarios due to their rapid strength development, excellent durability, and field workability. They offer a promising solution for improving the efficiency and reliability of emergency repair operations on various pavement types.

This study presented a laboratory-scale investigation on the workability and mechanical properties of CBPC composites with varying mix proportions. Nevertheless, further research is required to ensure practical applicability in field conditions. Future studies should address the influence of coarse aggregate incorporation with respect to cost-effectiveness, the material behavior under diverse casting environments (e.g., temperature and humidity), and the long-term durability under environmental and mechanical stresses. Such efforts are expected to provide more conclusive evidence for the applicability of CBPC composites in broader infrastructure contexts.

## Figures and Tables

**Table 1:** Experimental plan for material mixing.

Test	Specimens	P:M	W/B (%)	R (%)	F (%)	F-M (cm^2^/g)	Curing Period	Measurement Items
1	W/B20-F00-M2290	1:1.5	20	1	-	2290	15 min., 30 min., 60 min., 1 Day, 3 Days, 7 Days, 14 Days, 28 Days	(1) Flow table; KS L 5111 [23] (2) Hardening time (3) Compressive strength; KS L 5105 [24] (4) Flexural strength; KS F 2408 [25] (5) Adhesive strength, KS F 4042 [26] (6) Length change, KS F 4042 [27] (7) Surface finish performance (8) SEM (Scanning Electron Microscope; JSM-6390, Jeol Ltd., Tokyo, Japan)
	W/B25-F00-M2290		25					
	W/B30-F00-M2290		30					
	W/B35-F00-M2290		35					
	W/B40-F00-M2290		40					
2	W/B25-F10-M2290		25	1	10	2290		
	W/B25-F20-M2290				20			
	W/B25-F30-M2290				30			
	W/B25-F40-M2290				40			
	W/B25-F50-M2290				50			
3	W/B25-F10-M570		25	-	10	570 2970 3640		
	W/B25-F10-M2970							
	W/B25-F10-M3640							

R: Retarder (borax), F: Filler (Sand), F-M: Fineness of phosphate (KH_2_PO_4_).

**Table 2:** Composition of KH_2_PO_4_.

Appearance	Purity	pH (1% sol’n)	Sulfate (SO_4_)	Iron(Fe)
White Crystal	98.0% up	4.2–4.7%	0.02% max	0.01% max

**Table 3:** Composition of MgO (powder type).

Mg	Al	Si	P	S	Ti	Mn	Fe	Co	Cu	Zn	Sn
86.3	0.66	2.69	1.08	0.01	0.02	0.23	3.26	0.02	0.01	0.03	0.03

**Table 4:** Material properties of borax.

Borax	Density	Sodium Borate	Solubility	Melting Point
Na_2_B_4_O_7_·10H_2_O	1.73 g/cm^3^	15.0%	4.7%	743 °C

**Table 5:** Physical Properties of Used Fillers (Sand; Granite-Based Artificial Sand).

Classification	No. 3 Sand	No. 5 Sand	No. 6 Sand
Particle size (mm)	0.8–0.6	0.3–0.2	0.2–0.15
Grain strength (%)	88–92	90–94	91–96
Abrasion loss (%)	1.43	1.39	1.23
Water absorption (%)	2.2	2.3	2.6
Density (g/cm^3^)	2.51	2.43	2.6

**Table 6:** Mix proportions for field application.

	W/B	P:M	MKP (%)	Filler (%)			
				No. 3 Sand	No. 5 Sand	No. 6 Sand	Stone Powder
For Concrete	30	1:1.5	50	-	25	15	10
For Asphalt	46			7	38	15	10

## Data Availability

The original contributions presented in this study are included in the article. Further inquiries can be directed to the corresponding author.
